# Supplementary figures and images for: Fluorescent secreted bacterial effectors reveal active intravacuolar proliferation of Listeria monocytogenes in epithelial cells
Source: PLoS Pathog. 2020 Oct 12;16(10):e1009001. doi: 10.1371/journal.ppat.1009001 (PMC7580998; doi:10.1371/journal.ppat.1009001)

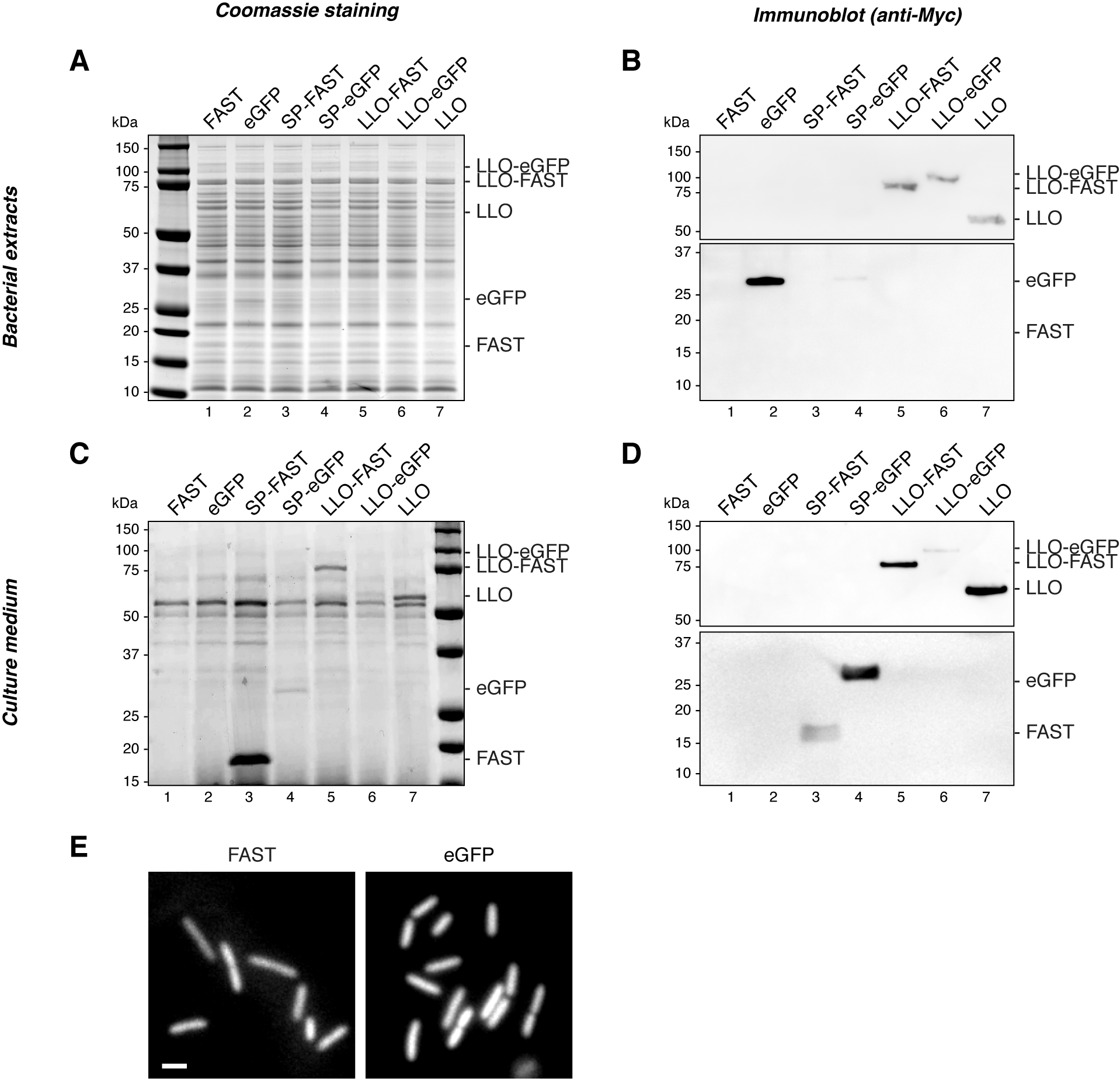

Supplement: S1 Fig — Protein production and secretion of Myc-tagged fusion proteins for each one of the constructs described in Fig 1A (constitutive expression from an integrated pAD vector) was assessed by colloidal Coomassie staining (A, C) and immunoblotting with anti-Myc antibodies (B, D) of bacterial total extracts (A, B) and culture supernatant fractions (C, D) from 16-h cultures in BHI, separated by SDS-PAGE. (E) Epifluorescence microscopy observation of strains producing non-secreted FAST or eGFP. Scale bar, 2 μm. Most Myc-tagged protein constructs were detected by immunoblotting in the corresponding bacterial pellet fraction, indicating that transgenes were expressed, even though in varying amounts (B, lanes 2, 4–7). Constructs harbouring the LLO SP or full-length LLO were recovered in bacterial supernatants (C, D, lanes 3–7), suggesting that the SP of LLO promoted Sec-dependent export of not only of FAST or FAST-tagged proteins, but also of eGFP-fusion proteins. The secretion of eGFP-tagged proteins seemed less efficient than that of FAST-tagged protein (C, compare lane 3 with 4; D, compare lane 5 with 6), consistent with previous reports that eGFP is a poor substrate for Sec-dependent secretion[18]. Constructs devoid of signal peptides were not detected in supernatant fractions (C, D, lanes 1–2), arguing against the release of proteins into the culture medium due to bacterial lysis. For technical reasons likely due to the small size of FAST-Myc (15 kDa), it was not or barely detected by immunoblotting (B, D, lanes 1, 3); nevertheless, a strong signal corresponding to this polypeptide was visible on Coomassie-stained gels of the supernatant fractions, attesting of its secretion (C, lane 3). For bacterial pellet fractions (A, lanes 1, 3), signal from other proteins masked possible bands from that polypeptide; however, observation in microscopy (E) confirmed the non-secreted form of FAST was also produced. (TIF) [file ppat.1009001.s001.tif]

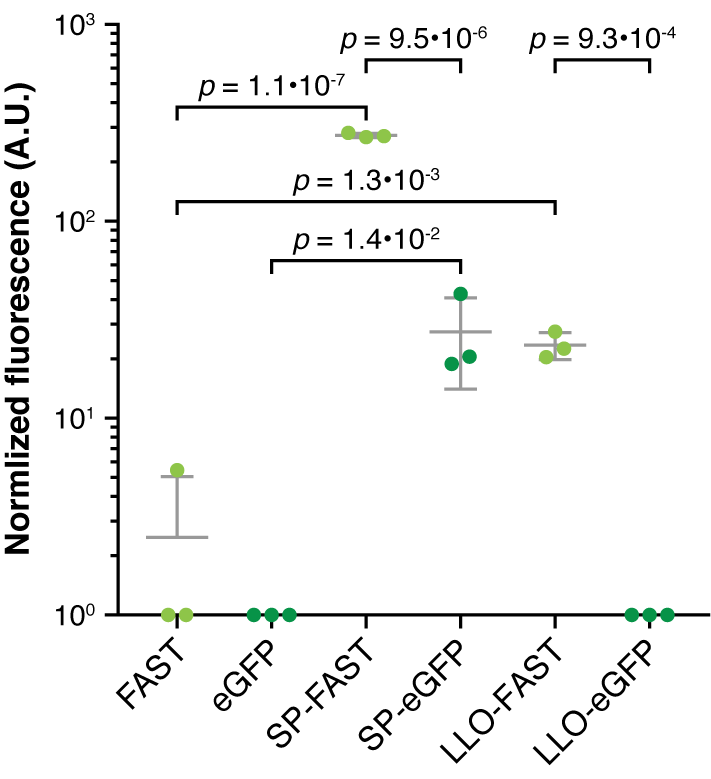

Supplement: S2 Fig — Six Lm strains expressing FAST- or eGFP-tagged proteins were cultured in LSM, then fluorescence intensities were measured on the filtered supernatants of each culture in presence of 5 μM HBR-3,5DM. For normalisation between FAST and eGFP signals, intensities were expressed in arbitrary units where 100 A.U. corresponds, for each reporter, to the intrabacterial fluorescence emitted by a suspension of equal volume of Lm (OD600nm = 1) that expresses constitutively either non-secreted FAST or eGFP under the PHYPER promoter. Residual fluorescence measured in the culture medium of strains producing non-secreted FAST or eGFP represents bacterial lysis. All values below 1 were considered below the detection limit for this experiment, and plotted as 1 (i.e. 100). Normalized values, means and standard deviations from three independent experiments were plotted. p-values represent the results of two-tailed Student’s t-tests with equal variance assumption. Source data are provided in S3 Table. (TIF) [file ppat.1009001.s002.tif]

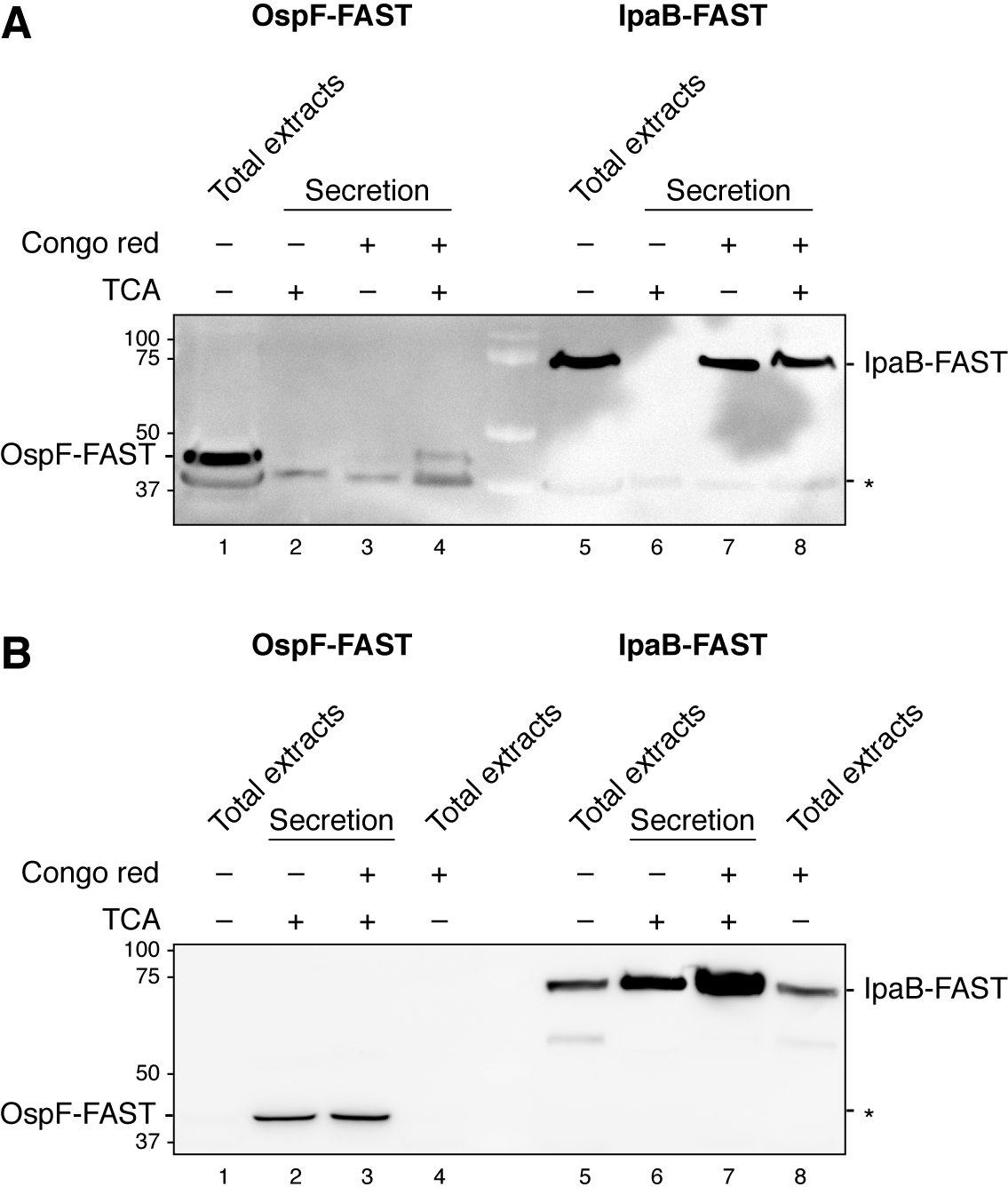

Supplement: S3 Fig — Protein production and secretion of Myc-tagged fusion proteins for each one of the constructs described in Fig 1C (constitutive expression from a pSU2.1rp vector) was assessed by immunoblotting with anti-Myc antibodies of bacterial total extracts culture supernatant fractions, with or without induction of secretion by the T3SS using Congo red, and with or without TCA precipitation in order to concentrate samples. (A) Samples from wild type M90T Sf. (B) Samples from M90T ΔipaD, in which T3SS secretion is constitutive. *, non-specific band. (TIF) [file ppat.1009001.s003.tif]

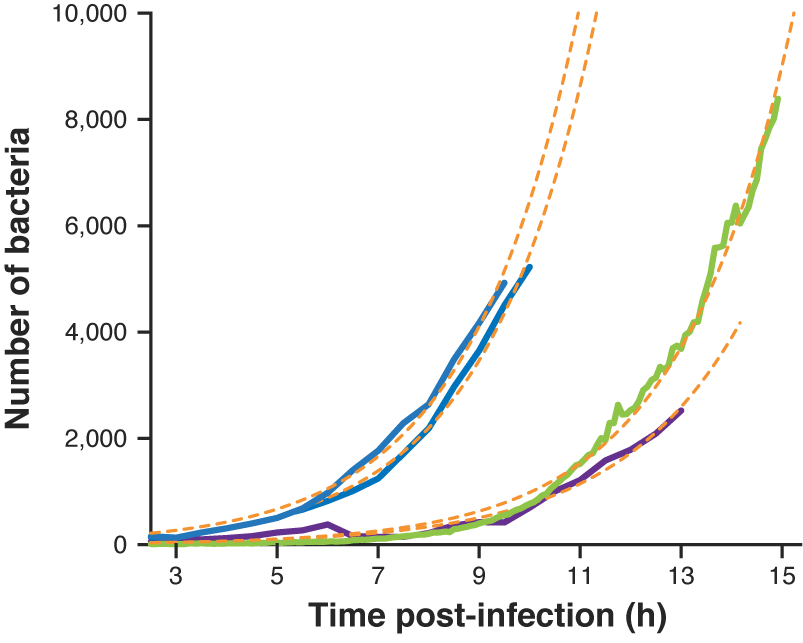

Supplement: S4 Fig — Dynamics of the total intracellular bacterial population were measured by segmentation of mCherry-labelled bacteria, in control wells recorded in parallel to the accumulation of SP-FAST in the cytoplasm (Fig 2). To get an estimate of the number of bacteria in each field, the total volume occupied by bacteria (the number of voxels that were labelled with mCherry) was divided by the average size of bacteria (32 voxels). Each colour represents an independent biological replicate (in blue, two wells were recorded in the same experiment). The exponential fit associated to each growth curve is displayed as orange dashed lines. (TIF) [file ppat.1009001.s004.tif]

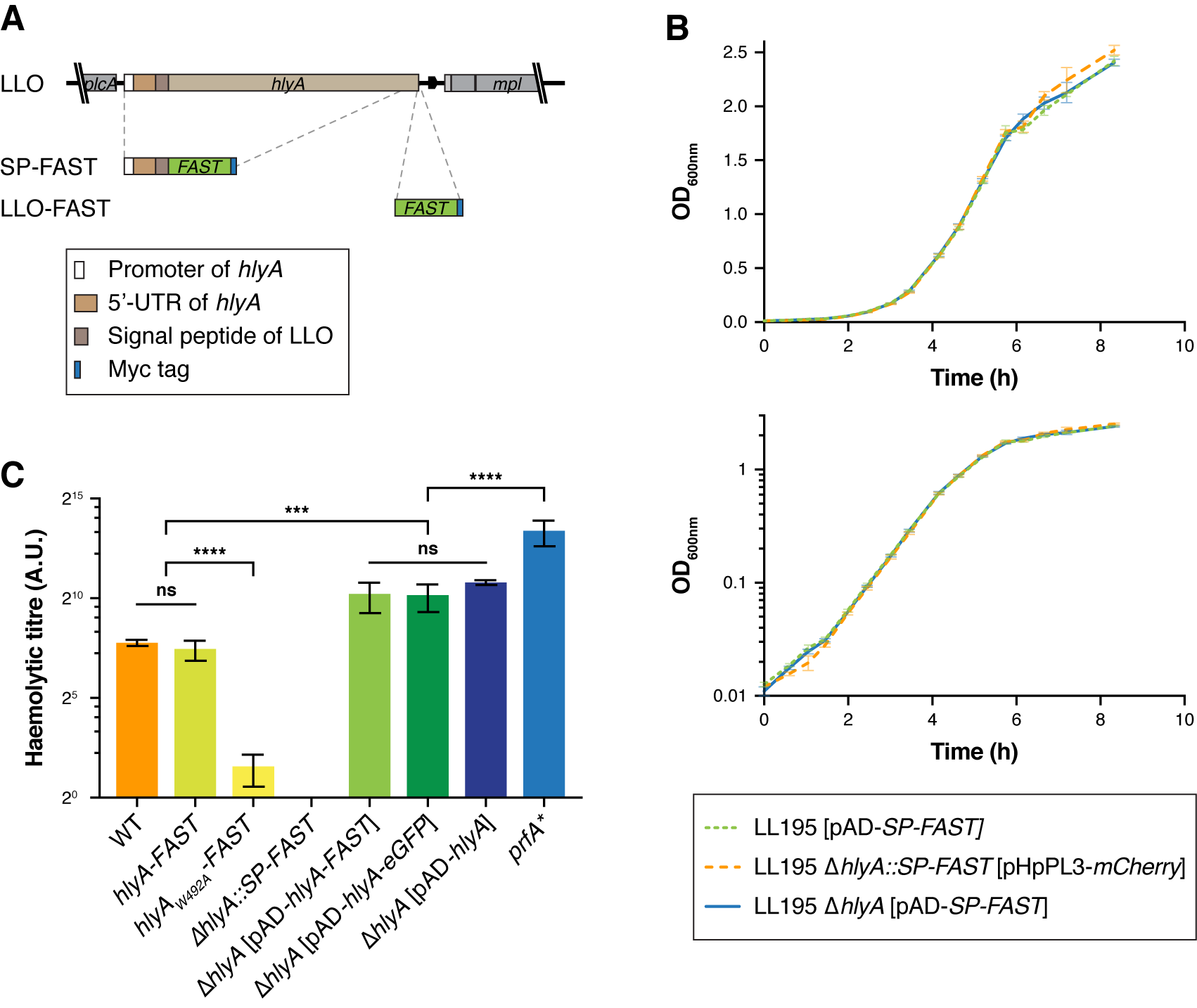

Supplement: S5 Fig — (A) Diagram of allelic replacement of hlyA (encoding LLO) at its chromosomal locus by a cassette expressing SP-FAST under the endogenous hlyA promoter (ΔhlyA::SP-FAST), and of in-frame C-terminal tagging of LLO with FAST (hlyA-FAST). (B) Growth curves of three Lm LL195 strains harbouring pPL2-derived vectors, at 37°C in BHI. No differences in growth rates were detected, regardless of the pPL2-derived plasmid that was integrated at the tRNAArg locus (pAD-SP-FAST or pHpPL3-mCherry) and of the genetic modification carried out at the hlyA locus (ΔhlyA or ΔhlyA::SP-FAST). Curves represent the average and standard deviation of technical triplicates, displayed in linear scale (top) or in semi-log scale (bottom). (C) Haemolytic properties of the Lm strains producing FAST–or eGFP–tagged LLO fusions used in this study. The haemolytic titre measured for the strain where LLO was C-terminally tagged with FAST-Myc at the hlyA locus (hlyA-FAST) did not differ from that of the WT Lm strain. The haemolytic titre of all ΔhlyA strains was null (here, only ΔhlyA::SP-FAST was plotted). Haemolytic titres were enhanced for ΔhlyA deletion strains that had been complemented by integrative pAD plasmids harbouring hlyA fusion genes under control of the constitutive PHYPER promoter. Fusion with FAST-Myc or eGFP-Myc (pAD-hlyA-FAST or -eGFP) did not affect haemolytic properties, compared to a simple fusion with Myc (pAD-hlyA). None of these strains reached the intense haemolytic properties of the prfA* strain (48.9-fold above the WT strain), for which the expression of Lm virulence genes (including hlyA) is deregulated, due to the constitutive activity of the transcriptional activator PrfA [22]. The average haemolytic titres and standard deviations from three independent experiments were plotted. Two-way ANOVA on log2-transformed haemolytic titres followed by post-hoc Tukey’s test was used for statistical testing between conditions. ns, non-significant; ***, p < 10−3, ****, p < 10−4. Source data [file ppat.1009001.s005.tif]

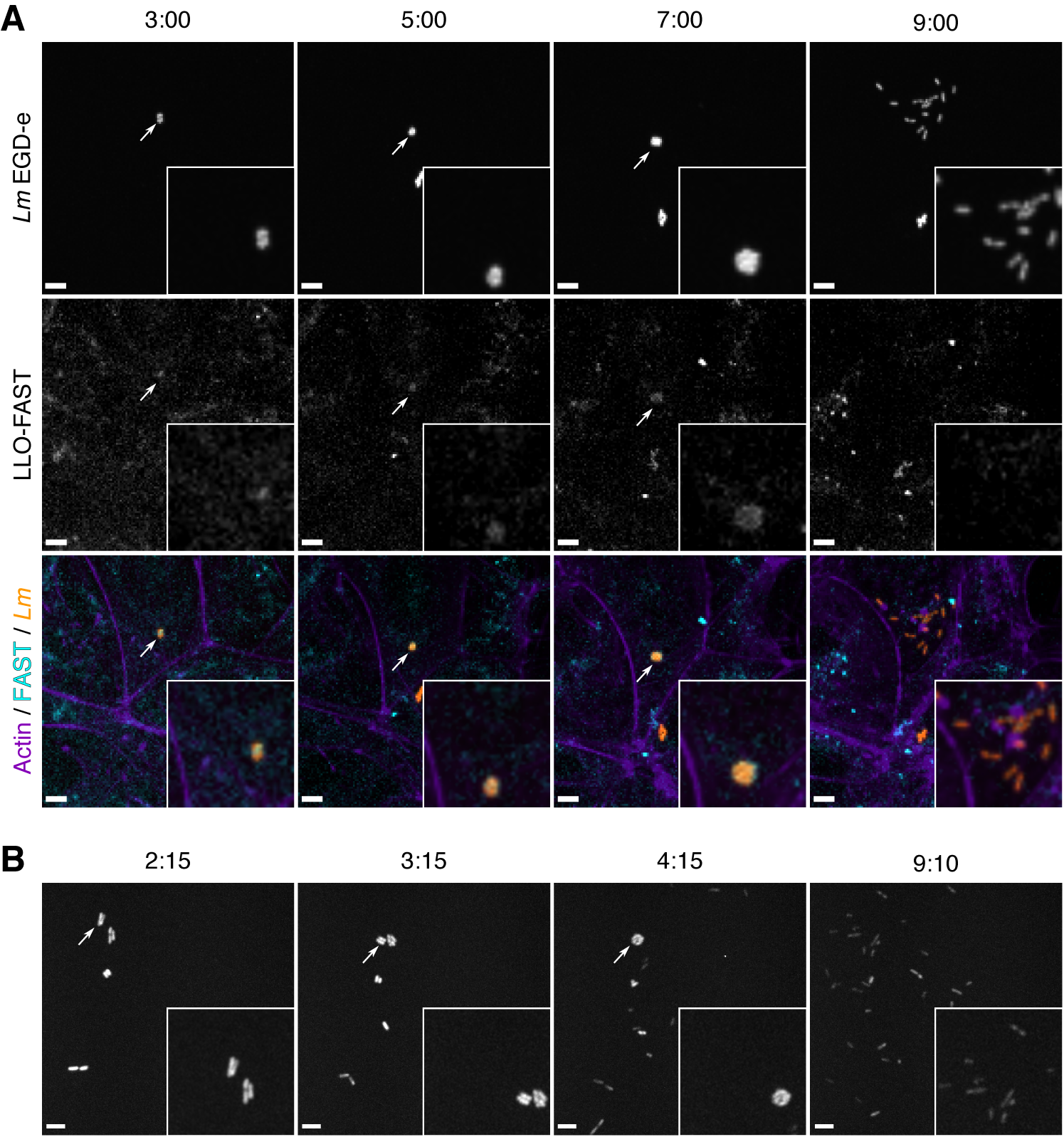

Supplement: S6 Fig — (A) LoVo cells infected with Lm EGD-e expressing both mCherry and LLO-FAST were observed between 2 and 8 h post-infection by spinning disk confocal microscopy. On the merged image, LLO-FAST is in cyan, mCherry is in orange, and SiR-actin is in purple. (B) Time-course of replication of mCherry-expressing Lm LL195 inside a vacuole, observed in the Caco-2 cell line. (A, B) Scale bars, 5 μm; timescale, h:min. (TIF) [file ppat.1009001.s006.tif]

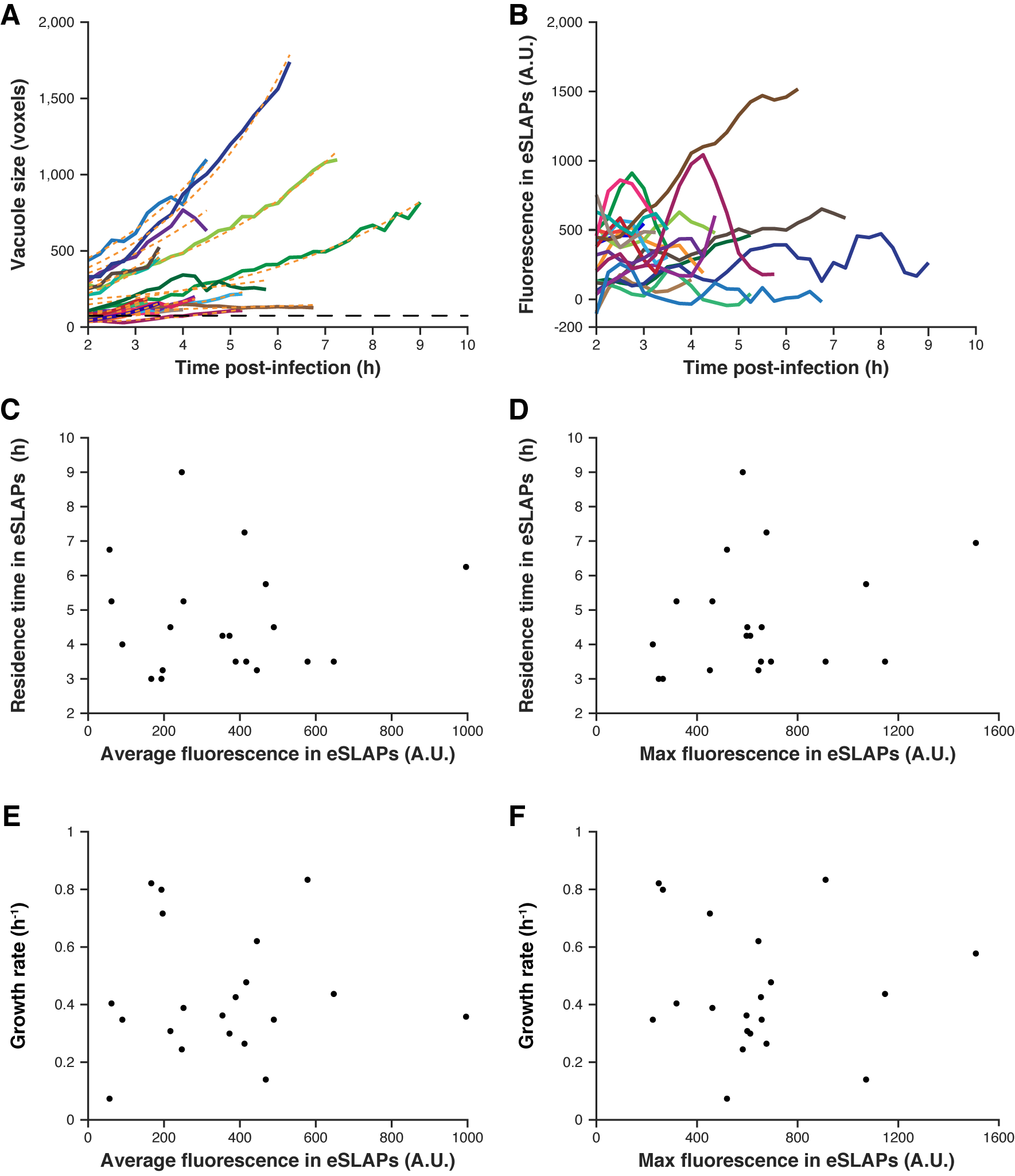

Supplement: S7 Fig — LoVo cells were infected with Lm carrying an integrated pHpPL3-mCherry plasmid and secreting FAST-LLO due to an in-frame C-terminal fusion with FAST at the hlyA locus. (A) Number of bacteria inside eSLAPs over time. mCherry signals allowed the segmentation of bacteria and their counting. The exponential fit associated to each growth curve is displayed as orange dashed lines. The black horizontal dashed line represents the volume of one average doubling event since the first frame. (B) Quantification of the fluorescence over time in the FAST channel, which reports for the concentration of LLO-FAST in eSLAPs. (C-F) Correlation between the fluorescence generated by LLO-FAST in eSLAPs and either the time residence time or the growth rate in these compartments. The average intensity of fluorescence generated by the secretion of LLO-FAST (C, E) and the maximum intensity of LLO-FAST fluorescence (D, F) were extracted for each eSLAP (n = 21) and correlated with the duration of this compartment since the beginning of acquisition (C, D) or with the growth rate of bacteria in this compartment, defined by the rate of increase of the size of the mCherry-labelled volume occupied by intravacuolar bacteria (E, F). (TIF) [file ppat.1009001.s007.tif]

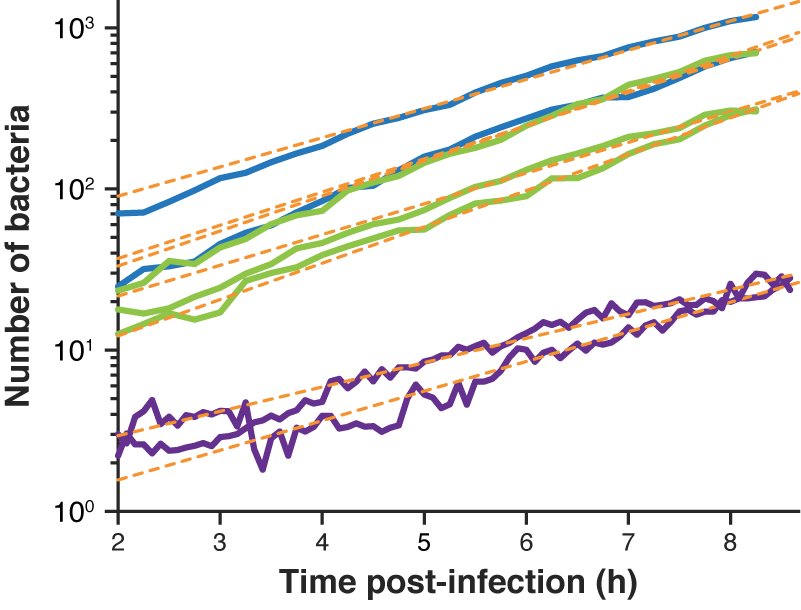

Supplement: S8 Fig — The number of mCherry-labelled bacteria was determined by segmenting the volume they occupied, as in S4 Fig. Each colour represents an independent biological replicate. Curves of the same colour represent technical replicates. The exponential fit (linear fit in semi-log scale) associated to each growth curve is displayed as orange dashed lines. (TIF) [file ppat.1009001.s008.tif]

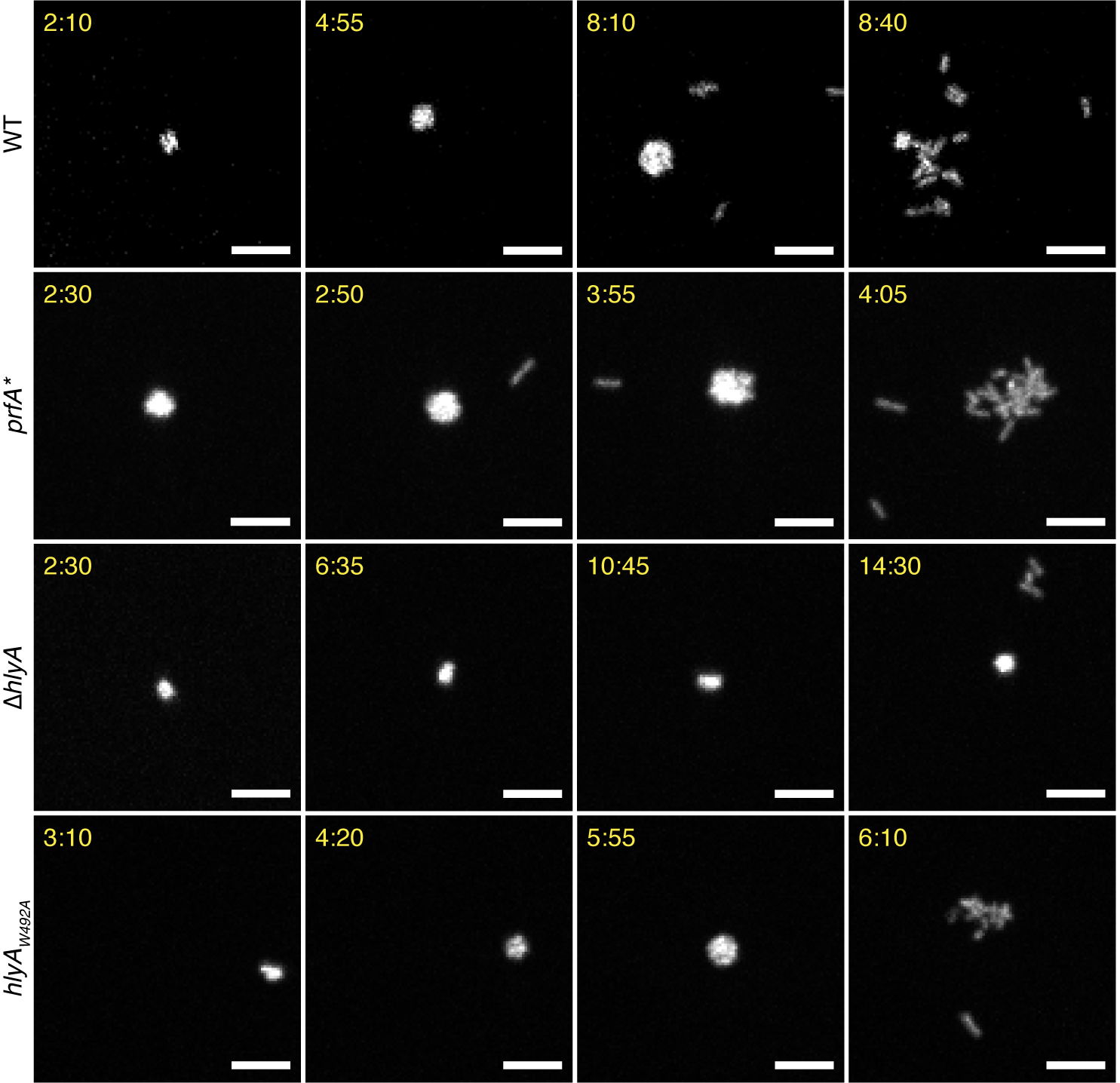

Supplement: S9 Fig — The fluorescent signal of mCherry expressed constitutively was used to locate bacteria. Bacteria that were confined in eSLAPs were packed in a spherical configuration and observed as large spots on fluorescence images. When the vacuole ruptured, membrane tension was released and bacteria dispersed into the cytoplasm. Scale bars, 5 μm; timescale, h:min. (TIF) [file ppat.1009001.s009.tif]

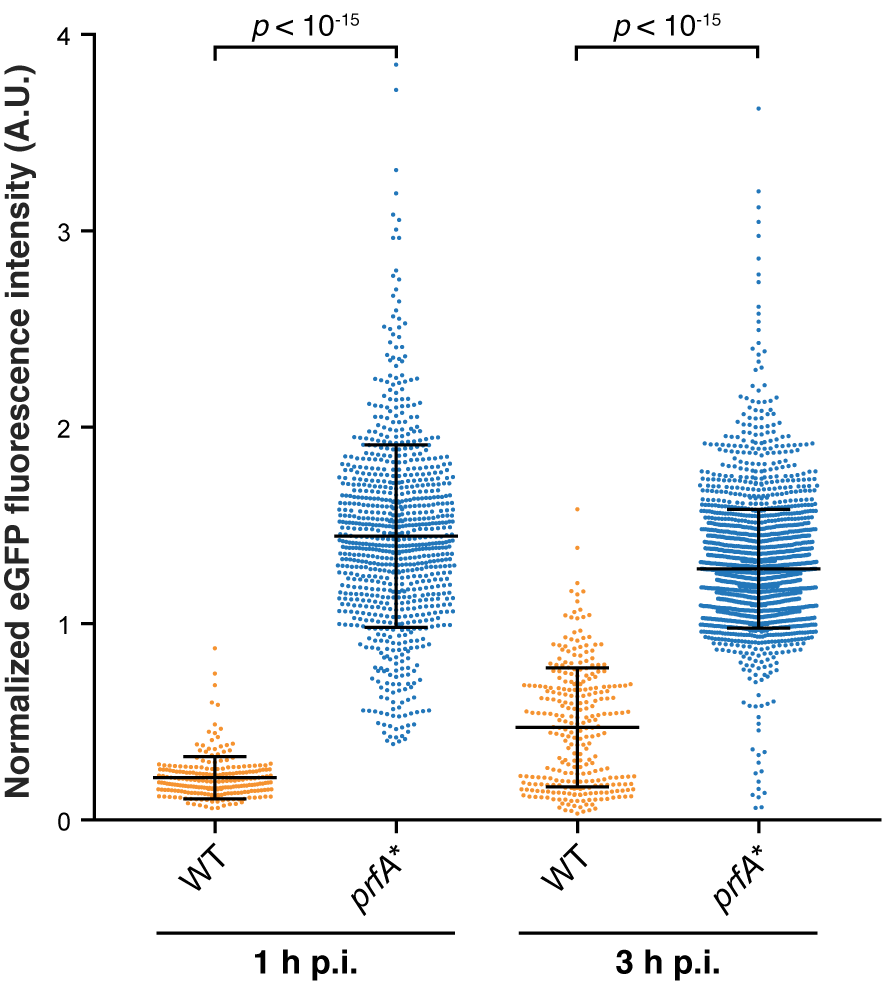

Supplement: S10 Fig — LoVo cells infected with either WT or prfA* EGD-e Lm strains, where eGFP was in transcriptional fusion with the promoter of hlyA by chromosomal allelic replacement (ΔhlyA::eGFP), and co-expressing mCherry. Data represent the ratio of eGFP to mCherry signals for each segmented bacterium. The number of analysed bacteria per condition was n = 234 for WT and n = 846 for prfA* bacteria at 1 h p.i.; n = 289 for WT and n = 2,124 for prfA* bacteria at 3 h p.i. Means and standard deviations are represented in black solid lines. Note that the distribution of intensities was bimodal for the WT strain at 3 h p.i., likely reflecting the induction of PhlyA in some, but not all of the bacteria at this stage. p-values indicate the results of Kruskal-Wallis non-parametric test followed by Dunn’s correction for multiple testing. Source data are provided in S8 Table. (TIF) [file ppat.1009001.s010.tif]

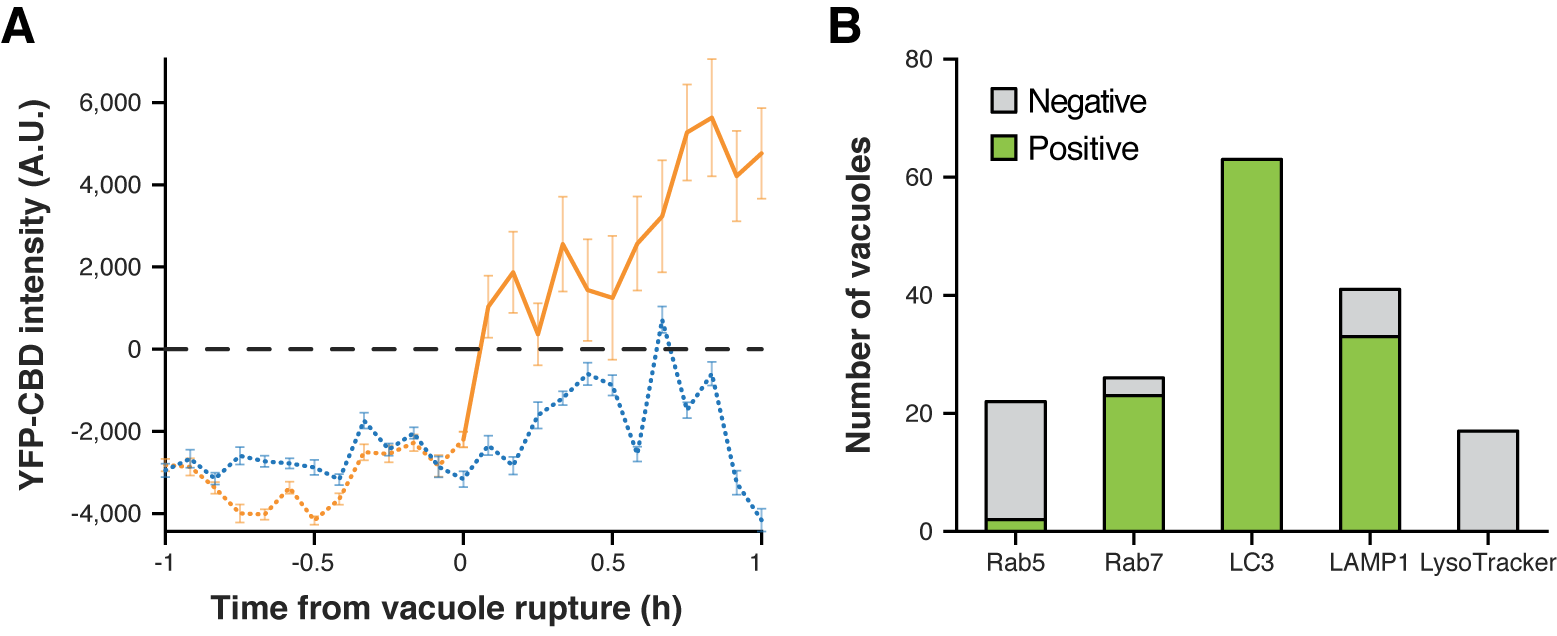

Supplement: S11 Fig — (A) YFP fluorescence intensity (reporting for the exposure of Lm to the host cytoplasm) was measured in bacteria before (dotted lines) or after (solid line) their release from eSLAPs, in the cell shown in Fig 5A and S5 Movie. The average background signal from the cytosol was subtracted and is represented as a black dashed line at 0 A.U. The orange trace displays the fate of a vacuole (dotted line) that ruptured at time 0 and released 11 bacteria (solid line) into the host cytosol. The blue trace represents a control vacuole in the same cell that did not rupture over the same time-course. Negative values correspond to signals below cytosolic levels, indicating that YFP-CBD was excluded from eSLAPs when they formed. In contrast, when bacteria were exposed to the host cytosol, they became positively stained. Error bars indicate the standard error of the mean. (B) The co-localisation of Rab5, Rab7, LC3 and LAMP1 with eSLAPs containing mCherry-labelled bacteria (immunofluorescence), or of LysoTracker Deep-red with GFP-labelled bacteria (live imaging), was assessed on infected LoVo cells at 3 h p.i. The occurrence of co-localisation events was 9% for Rab5 (2/22), 88,5% for Rab7 (23/26), 100% for LC3 (63/63), 80,5% for LAMP1 (33/41) and null for LysoTracker (0/17). Source data are provided in S9 Table. (TIF) [file ppat.1009001.s011.tif]

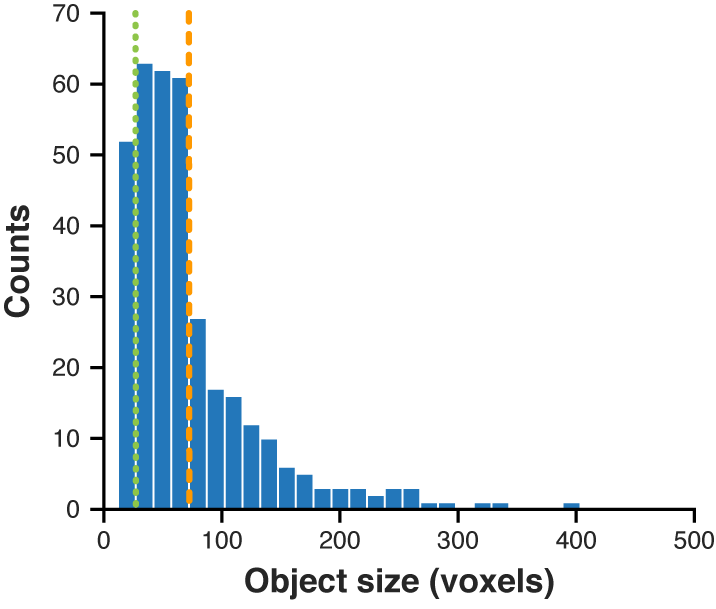

Supplement: S12 Fig — The first bin of the distribution (left of the green dotted line) corresponds to objects smaller than the size of bacteria that were discarded when counting the number of entry events. The bins between the green dotted and orange dashed lines correspond to single bacteria, the size of which was in the range of 32 to 64 voxels. The orange dashed line marks the limit between objects that correspond to individual bacteria (left) and clusters of bacteria (right). The mean of the distribution (n = 354 objects from 15 pooled experiments) was equal to 75 voxels. (TIF) [file ppat.1009001.s012.tif]
