# Supplementary material for: Fluorescent secreted bacterial effectors reveal active intravacuolar proliferation of Listeria monocytogenes in epithelial cells
Source: PLoS Pathog. 2020 Oct 12;16(10):e1009001. doi: 10.1371/journal.ppat.1009001 (PMC7580998; doi:10.1371/journal.ppat.1009001)
Supplement: S1 Text — (PDF) [file ppat.1009001.s018.pdf]

S1 Text. Synthetic gene fragments with codon-optimized sequences for expression of FAST fusions in (A) *Listeria* or (B) *Shigella*.

A ***Eag*I-5'-UTR$_{hlyA}$-SP$_{hlyA}$-FAST$_{Listeria}$-Myc-STOP-*Sal*I**

tgtgt***CGGCCG***ataaagcaagcatataatattgcgtttcatctttagaag
cgaatttcgccaatattataattatcaaaagagaggggtggcaaacggta
tttggcattattaggttaaaaaatgtagaaggagagtgaaaccc**ATG**AAA
AAAATAATGCTAGTTTTTATTACACTTATATTAGTTAGTCTACCAATTGC
GCAACAAACTGAAGCAAAGGATGAACATGTTGCTTTCGGTTCTGAAGATA
TCGAAAATACACTTGCAAAAATGGATGATGGTCAACTTGATGGCCTAGCA
TTCGGTGCTATTCAACTTGACGGCGACGGCAACATTCTTCAATACAACGC
GGCAGAAGGAGATATTACTGGTCGTGATCCAAAACAAGTAATTGGAAAAA
ACTTTTTCAAAGACGTTGCTCCAGGGACCGATAGCCCTGAATTCTATGGA
AAATTTAAAGAAGGTGTAGCTTCTGGCAATCTAAACACTATGTTCGAATG
GATGATCCCAACATCACGTGGACCAACAAAAGTTAAAGTTCATATGAAAA
AAGCACTAAGCGGAGATTCATATTGGGTTTTTGTTAAACGTGTAgaacaa
aaacttatcagcgaagaagatttaTAA***GTCGAC***ctcgagggggggcccgg
taccagctt

B ***Bam*HI-FAST$_{Shigella}$-Myc-STOP-*Xba*I**

ATG***ggatcc***GAACATGTTGCGTTCGGCAGCGAAGATATTGAGAACACCTT
AGCTAAAATGGACGATGGTCAGTTAGACGGCCTGGCTTTTGGTGCGATCC
AGCTGGACGGCGACGGCAACATCCTGCAGTACAACGCGGCTGAAGGTGAT
ATCACCGGTCGCGATCCGAAACAGGTGATCGGCAAAAACTTCTTCAAAGA
CGTTGCGCCGGGTACTGACTCTCCGGAATTCTACGGTAAATTTAAAGAGG
GTGTCGCGTCCGGTAACCTGAACACCATGTTCGAATGGATGATCCCAACC
TCCCGCGGCCCAACCAAAGTTAAAGTCCACATGAAAAAAGCACTGAGCGG
CGATTCTTATTGGGTGTTCGTTAAACGTGTTgaacagaaactgatctctg
aggaagatctgTAA***tctaga***gtcg
