# Supplementary material for: Fluorescent secreted bacterial effectors reveal active intravacuolar proliferation of Listeria monocytogenes in epithelial cells
Source: PLoS Pathog. 2020 Oct 12;16(10):e1009001. doi: 10.1371/journal.ppat.1009001 (PMC7580998; doi:10.1371/journal.ppat.1009001)
Supplement: Attachment — Submitted filename: Cover_Letter_V2.pdf [file ppat.1009001.s028.pdf]

**Alice Lebreton**

Group *Bacterial Infection & RNA Destiny*
Institute of Biology of École normale supérieure (IBENS)
46 rue d'Ulm
75 005 Paris
France
alice.lebreton@ens.psl.eu
Tél : +33 1 44 32 34 62

Paris,
September 17 2020

Dear Editor,

Please find enclosed a revised version of our manuscript "**Fluorescent secreted bacterial effectors reveal active intravacuolar proliferation of *Listeria monocytogenes* in epithelial cells**". We thank the reviewers for their positive and constructive comments, which helped improve the quality of the manuscript. We addressed reviewer 2's comments and carried out the suggested experiments, as detailed below in our point-by-point response. Also, we decided to slightly change the title of the revised manuscript, to account for the cellular context and to highlight the main difference between SLAPs and eSLAPs:  the rapidity of growth in vacuoles. We hope that the revisions now make the manuscript suitable for publication in *PLoS Pathogens*.

Yours sincerely,

Alice Lebreton and Nicolas Desprat.

## Point-by-point response to reviewers

We are grateful to the reviewers for their comments and constructive criticism on our manuscript. We feel that addressing them considerably strengthened our findings and improved the overall quality of our work.

## Reviewer #1

In this paper the authors developed a method to track secreted proteins in live microscopy. Authors show application of the new tracking system to two markedly different secretion systems in both Gram positive and Gram negative intracellular pathogens. They then used their tool to measure time of vacuole rupture in Listeria, which is an important step in Listeria infection cycle, freeing bacteria and allowing infection of the neighbouring cell. They observed that during the infection not all of the bacteria succeed in escaping the vacuole. This phenomenon, which occurs despite expression of the listeriolysin O (LLO) pore forming toxin, allows a subpopulation of Listeria to proliferate within these vacuoles reminiscent of macrophage SLAPs, from which they eventually escape. The eSLAP (for epithelial SLAP) therefore represents another replicative niche used by Listeria (and possibly other intracellular bacteria) to a colonize host cell. In addition, this piece of work provides an interesting tool that is relevant and potentially applicable to many bacterial organisms.

The work should therefore be of interest for a large community of bacteriologists. The paper is well written and easy to follow. Overall, this work is of outstanding quality and well supported by the results with appropriate controls and statistical analysis. I reviewed it earlier for another journal and the authors have addressed all the comments I had then. This is now a version that can be published without further delays.

Major comment — The paper is acceptable for publication as is, authors have taken on board all comments from a previous round of reviewing in which I happened to take part.

We thank Reviewer 1 for his/her review and hope that the additional changes we made to the manuscript will reinforce his/her opinion that this study deserves publication.

## Reviewer #2

This is an interesting work that describes the use of a FAST reporter to study the localization of LLO from Listeria in real time. Using a LLO-FAST reporter, the authors find that, in epithelial cell lines, a subpopulation of Listeria fails to escape from phagosomes and instead replicate in these structures in a manner dependent on LLO. They refer to phagosomes in epithelial cells that contain replicating Listeria as 'eSLAPS'.

This is a well performed study that provides new insight into the functions of LLO. I provide a few comments and questions below.

### Part II – Major Issues: Key Experiments Required for Acceptance

1. I could not find any information on the method of statistical analysis used in this study. In my view, it is critical to mention the statistical test used in each figure so that one can judge if the statistical analysis is appropriate.

The methods of statistical analysis used in this study are now documented in the figure legends (two-tailed Stutent's *t*-test for Figs 1B, 1D, 3C, S2 Fig; two-tailed Mann-Whitney's test for Fig 2B, two-way ANOVA for S5C Fig; Kruskal-Wallis for S10 Fig) and detailed in the methods when appropriate.

2. Does replication in eSLAPs require the pore-forming activity of LLO?

To address this question, we repeated the experiments that had been conducted using the Δ*hlyA* deletion strain, but using a point mutant of LLO-FAST with almost no haemolytic activity. Based on the literature, we selected the W492A point mutation, that was previously assessed to harbour 0.1% of the haemolytic activity of the WT strain in the L028 background (Michel *et al., Mol. Microbiol.* 1990, doi: 10.1111/j.1365-2958.1990.tb00578.x). This mutant has been most often considered haemolysis-null, and is one of the most affected, if not the most affected, described version of LLO to our knowledge. We introduced this point mutation in *hlyA-FAST* in the LL195 background and assessed the haemolytic titre of the resulting mutant bacteria (two independent clones were tested in independent replicates). Both clones retained ~1 to 3% of the haemolytic activity of the WT strain.

When using this strain, bacterial replication was still observed in eSLAPs, which might imply that LLO activity was not necessary for growth in eSLAPs. However, without a LLO point mutant showing absolutely no haemolytic activity, we cannot rule out that minute amounts of pore formation could be are sufficient to allow bacterial proliferation in vacuoles. We have added this information to the text, which reads: "To examine whether the activity of LLO as a pore-forming toxin was required for bacterial growth in eSLAPs, we carried out the same experiment using a strain with a W492A point mutation in LLO that had been previously described to almost abolish haemolytic activity [21]. The introduction of this mutation in chromosomal *hlyA*-FAST in the LL195 background resulted in a strain that retained ~1 to 3 % of the haemolytic titres of the isogenic WT strain (S5C Fig). These bacteria were still able to grow in vacuoles (S9 Fig), suggesting that either the pore-forming toxin activity of LLO was itself dispensable for allowing intravacuolar growth, or the residual activity of the toxin was enough to support growth."

As a side note, the possibility that low LLO activity would prolong *Lm* residence in vacuoles while allowing bacteria to replicate therein is consistent with the previous report by Birmingham *et al.* (*Nature* 2008, doi: 10.1038/nature06479) showing that reduced *hlyA* expression allowed slow replication in macrophage SLAPs. We have now mentioned this in the discussion. The minimal level of LLO activity or expression that would be needed to promote growth however would require more detailed analysis in the future.

3. Line 251. Just because the PrfA* mutant form of PrfA is constitutively activated in broth culture does not necessarily mean it is more active than wild-type PrfA in host cells, given that PrfA is activated in cells. Are there published data demonstrating that a PrfA* mutant stimulates higher expression of PrfA-dependent genes in host cells compared to wild-type bacteria? Although the images in Fig. S9 give the general impression that LLO-FAST accumulates to a greater extent in eSLAPs from PrfA* bacteria compared to wild-type bacteria, this apparent difference is not quantified. I would suggest quantification from several eSLAPs and the use of statistical analysis to determine if PrfA* does, in fact, cause increased accumulation of LLO-FAST.

Previous reports have investigated the intracellular expression of PrfA-dependent genes in WT and *prfA** strains. While none of them exactly corresponds to our conditions, we provide below an overview of what we considered the most relevant related studies:

**A.** Using a *lacZ* reporter in WT bacteria, Moors *et al.* (*Infect. Immun.* 1999, doi : 10.1128/IAI.67.1.131-139.1999) quantified the fold-induction of the activity of the $P_{hlyA}$ promoter between growth in broth and intracellular growth in J774 mouse macrophages. The expression of the *lacZ* reporter was induced 20-fold in intracellular bacteria at 5 h post-infection (p.i.), which was slightly lower than (but in a similar order of magnitude as) the 50-fold increase in haemolytic titres that we measured *in vitro* for the *prfA** strain compared with the WT strain. The same study reported that *actA* was up-regulated at much higher levels (226-fold), indicating variability in intracellular induction of the different PrfA-dependent genes.

**B.** Using a *gus* reporter, Shetron-Rama *et al.* (*Infect. Immun.* 2002, doi: 10.1128/IAI.70.3.1087–1096.2002) quantified the activity of the *actA* promoter (or of a chimeric promoter where part of the *actA* promoter was replaced by the PrfA-binding site of the *hlyA* promoter) in broth for WT or *prfA** bacteria , and in J774 cells at 5 h p.i for WT bacteria. They reported, that the activity of the $P_{actA}$

promoter was ~50-fold higher for WT bacteria in cells that for the *prfA** strain grown in broth (Fig. 6B). However, $P_{actA}$ promoter activity was not assessed for intracellular *prfA** bacteria. This study also shows that addition of charcoal in broth is able to enhance the activity of the $P_{actA}$ promoter even in a *prfA** strain (Fig. 6A), suggesting that even in bacteria harbouring the *prfA** mutation, the expression level of virulence genes remains linked to complex environmental factors, and can be further potentiated.

**C.** In a review published in 2011 in *Current Opinion in Microbiology*, de las Heras *et al.* (doi:10.1016/j.mib.2011.01.005) measured by RT-qPCR (Fig 4) the relative expression of *actA* for WT and *prfA** bacteria grown either in broth or in HeLa cells. In HeLa cells, the level of expression of *actA* was similar for WT and *prfA** bacteria. Unfortunately, we did not find in the text at which time post-infection these measurements were carried out in this work.

**D.** Deshayes *et al.* (*Mol. Microbiol.* 2012, doi:10.1111/j.1365-2958.2012.08121.x) compared the activities of the *plcA* and *actA* promoters by RT-qPCR in various strains of bacteria grown either in broth, or in HeLa cells (6 h p.i.). For both genes, expression levels in intracellular bacteria were in the same range for *prfA** or isogenic WT bacteria, suggesting as in **C** that at this time p.i. and for these genes, induction was maximal.

Based on $P_{actA}$ or $P_{hlyA}$ activities, the expression of PrfA-dependent genes by the WT strain was previously found to be in the same range as that of the *prfA** strain, due to the *in-vivo* activation of PrfA. However, these previous studies have not directly compared the expression level of *hlyA* between WT and *prfA** strains in cells. As suggested by **A** and **B,** distinct genes in different environmental contexts may respond differentially. In addition, most of the above-described experiments were carried out at rather delayed time-points p.i. (at least 6 h p.i.), in cellular models where most of the bacteria were cytosolic. We thus thought worthy to assess whether intracellular *prfA** bacteria did actually overexpress *hlyA* in our experimental model.

To this end, Reviewer 2 suggested quantifying LLO-FAST signals in S9 Fig. However, S9 Fig displays mCherry signals, which were used to segment vacuoles or bacteria. mCherry was constitutively expressed (chromosomically-integrated $P_{HYPER}$-*mCherry* fusion). We modified the legend or this figure in order to make it clearer. The *prfA** strain that was used in this experiment did not express *hlyA-FAST*, thus unfortunately LLO-FAST signal cannot be recorded from this experiment.

Measuring LLO-FAST in the vacuoles formed in the WT and *prfA** backgrounds is technically feasible. However, LLO-FAST signal in vacuoles is not directly proportional to *hlyA* expression, because the dynamics of LLO-FAST concentration in the vacuoles is influenced by several external factors like membrane leakage or protein degradation. Importantly, active mechanisms of LLO elimination in cells have been previously reported, mainly due to (*i*) the ubiquitin-proteasome pathway and (*ii*) its PEST sequence (reviewed in Nguyen *et al., Cell. Microbiol*. 2018, doi: 10.1111/cmi.12988). All these contributions to the intravacuolar dynamics of LLO-FAST would prevent us from directly relating LLO-FAST signals to the actual expression levels of *hlyA*, and thus from drawing clear-cut conclusions. Therefore, we addressed the reviewer's concern by using a reporter strain that directly informed on the activity of the $P_{hlyA}$ promoter in intracellular WT or *prfA** bacteria.

Reasoning that the effect of *hlyA* production on the stability of eSLAPs must operate since the very early beginning of vacuole formation, when most bacteria were still intravacuolar, we used a reporter strain where *eGFP* was expressed under control of the $P_{hlyA}$ promoter to assess the expression of *hlyA* in individual WT of *prfA** bacteria, at 1 h and 3 h p.i. This choice was also motivated by previous reports showing that maximal *hlyA* induction was reached in phagosomes, while maximal *actA* induction was reached in the cytosol (Bubert *et al., Mol. Gen. Genet.* 1999, doi:10.1007/pl00008633).

In our model system, while the intensity of eGFP fluorescence for the WT strain increased at 3 h p.i. (in part of the bacterial population, giving rise to a bimodal distribution) *versus* 1 h p.i., eGFP intensities

remained significantly lower than for the *prfA** strain, at both time-points. Hence even in host cells, the *prfA** strain was still overexpressing *hlyA* compared to the WT strain, at least in the first hours of infection.

We have added data about $P_{hlyA}$-*eGFP* expression in intracellular bacteria as S10 Fig, and included this information in the text, which now reads: "Using WT or *prfA** reporter strains where eGFP was inserted by allelic replacement under control of the endogenous $P_{hlyA}$ promoter, we quantified that eGFP expression was on average 6.7-fold higher in the *prfA** strain than in the WT strain at 1 h p.i., and 2.7-fold higher at 3 h p.i. (S10 Fig). In our experimental model, the *prfA** mutation thus also led to overexpression of *hlyA* by intracellular bacteria, at least in the first hours of infection when eSLAPs start being formed by intravacuolar bacteria."

### Part III – Minor Issues: Editorial and Data Presentation Modifications

1. Figure 2C. It is difficult for this referee to distinguish between the green points and the blue curve. I would suggest changing blue to another color. Actually, black might work fairly well.

We appreciate the reviewer's suggestion to enhance the readability of this panel. The line is now displayed in black as per the reviewer's suggestion. The figure legend and text were amended accordingly.

In line with this comment, we also updated the use of colours in other figures of the article, either to enhance readability, or to increase consistency in colour codes from figure to figure. Figure legends have been amended accordingly.

2. Line 191, 'significantly longer'; line 193 'a significant proportion'. I would avoid the use of 'significant' in these contexts, since statistical analysis did not seem to be performed on Fig. 3C or Fig. 3D. In a related matter, I wonder why statistical analysis was not carried out on Fig. 3D. It would seem to be important to determine if the difference between the WT and ΔhlyA strain is significant.

In the revised version, we carried out statistical testing on these sets of data, to support our statement that differences were significant. For Fig 3C, statistical testing for the null hypothesis between the two distributions by a two-tailed *t*-test returned $p = 0.0191$. Since this level of confidence is appropriate for this type of experiment; we kept the word "significant" in the first sentence. In the second sentence, "significant" had been mistakenly used instead of "consistent", which appears more appropriate. These sentences now read: "When using the Δ*hlyA::SP-FAST* strain, the distribution of residence times was significantly shifted compared to the WT ($p = 0.0191$). The median residence time was longer (21.1 ± 1.4 min) but remained of the same order of magnitude as for a strain producing LLO, confirming previous observations that *Lm* gained efficient access to the cytoplasm independently of LLO in epithelial cells [11]. Unexpectedly, a consistent proportion of the entry vacuoles lasted for more than one hour (12.0 % for the WT strain; 14.8 % for the Δ*hlyA* mutant), and intact vacuoles were still observed 3 h p.i. (4.6 % for the WT strain; 6.2 % for the Δ*hlyA* mutant) (Fig 3C)."

The method used for the calculation of the interpolated median is now also detailed in the methods section.

3. Fig. S7A. What do the dashed lines represent?

We have now indicated what the dashed lines represent as follows: "The exponential fit associated to each growth curve is displayed as orange dashed lines. The black horizontal dashed line represents the volume of one average doubling event since the first frame."

4. Fig. S7B. How does one account for the fluctuating in LLO-FAST signals in some of the vacuoles (Fig. S7B)? Is LLO-FAST being degraded and then re-synthesized?

Fluctuations of LLO-FAST signals in vacuoles result from a complex interplay between several parameters: synthesis, secretion, leakage and degradation (the latter being strongly influenced by the short half-life of LLO; see also our reply to Part II, point 3). We rephrased the sentence describing these fluctuations, which

now reads: "In some vacuoles, LLO-FAST accumulated linearly over time, while others displayed large-scale fluctuations in signals that may reflect variations in LLO synthesis, secretion, degradation, leakage from vacuole or membrane repair."

5. Line 248. Should be Fig. 4C (not Fig. 3C).

From the distributions of residence times in entry vacuole for the WT and *ΔhlyA* strains (Fig 3C), one can see that the tail of the distribution (long-term vacuoles) does not depend on LLO secretion. However, Reviewer 2 is right that Fig 4C also supports that statement. Hence, we now mentioned both Fig3C and Fig 4C as evidence that lasting vacuoles are observed with the *ΔhlyA* strain.

6. Line 251. What is meant by 'Symmetrically'?

We thank the reviewer for pointing this confusing word. It was now replaced with "Conversely, to assess the effects of increased LLO production".

7. Line 279 and Fig. 5A. To this referee, the evidence that eSLAP rupture precedes decoration of bacteria with YFP-CBD does not seem that compelling in this image. The CBD labeling seems quite weak and I'm not convinced that CBD-labeled bacteria appear in the same area of the cytosol that previously was occupied by the vacuolar structure labeled with Lm. Perhaps the authors could select a clearer set of images?

We acknowledge that this image was not the most compelling with regards to the labelling of cytosolic bacteria after escape from eSLAPs, likely due to a low transfection level of the chosen cell. We have now replaced the set of pictures and videos (Fig 5A, S5 Movie), where two eSLAPs formed in the same cell. eSLAPs did not display YFP-CBD labelling, while the bacteria clearly acquired labelling after accessing the cytosol. The intensity of the intravacuolar or cytosolic labelling of bacteria with YFP-CBD is quantified in S11A Fig.

## Data requirements

Please note that, as a condition of publication, PLOS' data policy requires that you make available all data used to draw the conclusions outlined in your manuscript. Data must be deposited in an appropriate repository, included within the body of the manuscript, or uploaded as supporting information. This includes all numerical values that were used to generate graphs, histograms etc.

In this revised version, we are providing supporting tables containing numerical values that were used to generate graphs, as per PLoS data policy.
